# *Aeromonas* spp. and Infectious Diarrhea, Hong Kong

**DOI:** 10.3201/eid1008.030518

**Published:** 2004-08

**Authors:** Stewart Siu-Wa Chan, King Cheung Ng

**Affiliations:** * The Chinese University of Hong Kong, Hong Kong

**Keywords:** letter, Aeromonas, infectious diarrhea, gastroenteritis

**To the Editor:** Vila et al. reported the prevalence of *Aeromonas* spp. associated with traveler’s diarrhea in Spain (1). Some of the patients described in this study had traveled to countries in Asia, such as Thailand and India. This report details the prevalence of this pathogen in patients with acute infectious diarrhea who were treated in emergency department settings in Hong Kong.

Over a 12-month period, we retrospectively studied all adult patients who showed clinical features of acute infectious diarrhea, were treated as outpatients with or without observation in the emergency department, and had a positive stool culture (2–4). Our data were collected at an urban university-affiliated hospital with 1,400 beds and an emergency department with an annual census of 190,000 patient visits. *Aeromonas* spp. were isolated from stool samples by standard culture procedures, which included introduction onto xylose lysine desoxycholate agar plate and thiosulphate citrate bile sucrose plate, and subsequent screening by triple iron sugar slant (acid butt with no H_2_S), positive oxidase, negative urease, fermentation of mannitol but not dulcitol and inositol, resistance to vibriostatic agent 0/129, and ability to grow at 0% NaCl. The main species of *Aeromonas* were identified by the differential biochemical reactions of gas production from D-glucose, arginine dihydrolase, ornithine and lysine decarboxylase; esculin hydrolysis; Voges Proskauer reaction; fermentation from arabinose, sucrose, mannitol, salacin, and D-sorbitol; and citrate and glycerol utilization (5).

Of 130 patients with positive stool cultures, *Aeromonas* spp. were isolated in 9 patients (6.9%), including *A. caviae* in 4 patients, *A. hydrophila* in 2 patients, and *A veronii* in 3 patients. The cases were not epidemiologically linked. In one of these isolates (*A. caviae*), another enteropathogen (*Vibrio parahemolyticus*) was also isolated. None of the patients reported recent travel abroad or to mainland China before treatment.

Our review of the clinical features of these nine patients found that the mean highest body temperature at the time of treatment or during the patient’s stay in the emergency department was 37.4°C (95% confidence interval [CI] 36.9–38.0). Two patients (both with *A. caviae* isolated) had temperatures >37.5°C. Bloody diarrhea was present in two patients (one with *A. veronii* and one with *A. caviae*). The mean number of unformed stools per day was 8.6 (95% CI 4.0–13.2). Abdominal pain in eight patients and vomiting in four patients was reported. Five patients required admission to the emergency department’s observation unit before discharge. Of these, four patients needed intravenous fluid therapy. Empiric ciprofloxacin was given to one patient with a temperature of 38.3°C. Stool culture results were available within 3 days for positive isolation of *Aeromonas*. All *Aeromonas* strains were susceptible to ciprofloxacin, cefotaxime, cotrimoxazole, and chloramphenicol, while two of nine isolates (one *A. caviae* strain and one *A. hydrophila* strain) were susceptible to ampicillin. All patients had recovered satisfactorily by the time stool culture results were available, and antimicrobial therapy was not necessary, except for the patient who was given ciprofloxacin empirically.

In conclusion, *Aeromonas* spp. are responsible for a small proportion of cases of bacterial gastroenteritis encountered in an urban emergency department setting in Hong Kong. Patients affected do not necessarily have a history of travel to a nonindustrialized region. In a substantial proportion of cases, the symptoms are severe enough to require intravenous fluid therapy and observation. However, symptoms generally would have resolved by the time the pathogen was isolated from stool culture. In contrast to the report of Vila et al., persistent diarrhea is uncommon, and antimicrobial therapy is usually unnecessary in our particular setting. *Aeromonas* spp. are susceptible to a wide range of antimicrobial drugs, except ampicillin. Whether empiric antimicrobial drugs given at the time of treatment would have significantly shortened the duration of the symptoms is not known.
